# Serum cortisol level and adrenal reserve as a predictor of patients’ outcome after successful cardiopulmonary resuscitation

**DOI:** 10.15171/jcvtr.2016.12

**Published:** 2016-06-28

**Authors:** Reza Mosaddegh, Nahid Kianmehr, Babak Mahshidfar, Zahra Rahmani, Hamed Aghdam, Mani Mofidi

**Affiliations:** ^1^Firoozgar Hospital, Iran University of Medical Sciences (IUMS), Tehran, Iran; ^2^Rasoul Akram Hospital, Iran University of Medical Sciences (IUMS), Tehran, Iran; ^3^Emergency Management Research Center, Iran University of Medical Sciences, Tehran, Iran

**Keywords:** Cardiopulmonary Arrest, Cardiopulmonary Resuscitation, Adrenal Insufficiency, Return of Spontaneous Circulation, Cortisol Level, Pituitary-Adrenal Axis

## Abstract

***Introduction:*** It is thought that pituitary-adrenal axis has a fundamental role in outcome of cardiopulmonary arrest (CPA). This study designed to evaluate the correlation between adrenal reserve and post-resuscitation outcome.

***Methods:*** In this clinical trial study, 52 consecutive patients with CPA were enrolled in two emergency departments (EDs) over a 3-month period. Plasma cortisol level was measured at the beginning of CPR. Intravenous adrenocorticotropic hormone (ACTH) stimulation test was carried out after successful CPR, and blood samples were taken at 30 and 60 minutes, and 24 hours thereafter. Patients were divided into two groups: in-hospital death or hospital discharge.

***Results:*** In patients who died, baseline and post-ACTH serum cortisol after 30 and 60 minutes and 24 hours were higher than patients who discharged from the hospital, but it was not statistically significant except to that of minute 60 (*P*=0.49). A model of multivariate logistic regression analysis showed that age and need for vasopressor infusion correlated with mortality.

***Conclusion:*** Current study could not show the statistically significant difference in initial and post-ACTH serum cortisol levels between survivor and non-survivor patients with cardiac arrest who had initial successful CPR, except to that of minute 60.

## Introduction

According to American Heart Association’s National Registry of cardiopulmonary resuscitation, survival to hospital discharge is 17.6% after in-hospital cardiopulmonary arrest (CPA) (for all rhythms).^1^

Time of circulation recovery has a paramount role in post resuscitation outcome. However, some secondary pathogenic processes cause organs dysfunction and influence patient outcome.^2-4^

Among these factors, RAI has been associated with poor prognosis in the critically ill patients.^5^ Any stress can stimulate the hypothalamic–pituitary–adrenal (HPA) axis and therefore change serum cortisol level.^6^

In Tsai clinical trial, patients who received hydrocortisone during CPR achieved higher ROSC rates.^7^

Hékimian et al^8^ reported that patients with successful CPR may die because of early refractory shock due to impaired adrenal reserve. In contrast, de Jong et al^9^ showed that pituitary-adrenal axis is more active in non-survivors than survivors of CPA and concluded that there is no association between RAI and death; hence, hydrocortisone prescription is not necessary.

The aim of the current study was to evaluate the status of HPA axis function by serum cortisol assay in victims of CPA with successful CPR. Results were analyzed in relation to death, survival, and hospital discharge of patients.

## Materials and Methods

We conducted a clinical trial which was approved by medical ethics committee of Tehran University of Medical Sciences (TUMS). It was registered in Iranian Registry of Clinical Trials (IRCT201111092337N5). Fifty-two patients, who had successful CPR in two academic emergency departments (EDs) since January 2012 to March 2012, were enrolled in the study. All of the patients experienced CPA in ED.

The exclusion criteria were: age less than 18, end stages of chronic diseases like cancer, and steroid use prior to CPA. The patients were resuscitated by emergency medicine residents by supervision of emergency medicine faculty according to American Heart Association guideline 2010. The ROSC was confirmed if the patients showed central or peripheral pulses after each 2 minutes cycle of CPR.

### Study protocol

Plasma cortisol was measured at the beginning of the CPR process. Cosyntropin test was carried out using 0.1 mg (40 units) intravenous adrenocorticotropic hormone (ACTH) as a bolus injection after a successful CPR. Blood samples were taken at 30 and 60 minutes and also 24 hours after ROSC. Serum cortisol was measured using an immune-enzymetric assay (Vidas-cortisol; Biomérieux, Lyon, France). The detection limit was considered 0.2 μg/dL. Response to cortisol (Δmax) was defined as the difference between the baseline and levels in the abovementioned intervals.

We used low dose test because of its better accuracy in diagnosis of subtle deficiency.^10^ Non-responders were defined as those having less than 9 μg/dL increase in cortisol level which was named as RAI.^11^ A primary outcome was patients’ outcome as in-hospital death or hospital discharge according to the status of HPA axis function.

### Statistical analysis

Sample size was 52 cases that calculated by Cochran formulas, Z: 1.96, p & q: 0.5, the acceptable margin of error: 5% and a 95% CI. The power of the study was 80%.

Results were presented as mean ± standard deviation (SD) for quantitative variables and were summarized by absolute frequencies and percentages for categorical variables. Categorical variables were compared using chi-square test. Quantitative variables were also compared with *t* test. Multiple variable logistic regression model analyses were used to estimate the odds ratios of outcome parameters (95% CIs).

Relations between two continuous variables were analyzed using the Spearman rank correlation test. Statistical analyses were done using SPSS 17.0 (SPSS Inc., Chicago, IL, USA). *P* values of 0.05 or less were considered statistically significant.

## Results

Fifty-two patients with successful CPR after CPA were included in the study during a 3-month period. The median age of patients was 68.78 (SD: ± 2.21) years. 30 (57.7%) patients were men. The mean interval time since arrest to CPR and arrest to ROSC were 7.32 ± 0.89 and 21.75 ± 14.04 minutes, respectively. All the patients experienced in hospital CPA in ED. CPA had cardiac origin in 10 of 41 dead and 9 of 11 discharged patients, respectively (*P *= 0.01).

Forty-one (78.8%) patients died; the cause of death was assumed early refractory shock in 13 and late organ dysfunction in 28 of them. Fifty-two samples were obtained 30 and 60 minutes after ACTH stimulation test, compared to 39 samples after 24 hours. Patients’ characteristics are shown in Table 1.

**Table 1:** Patients’ characteristics

**Characteristics**	**Non-survivors (n=41)**	**Survivors (n=11)**	**P value**
Age (years)	69.11 ± 3.43	63.01 ± 2.77	0.03^*^
Sex (males %)	23 (56.09)	7 (63.63)	0.49
CPR duration (min)	22.12 ± 3.22	19.32 ± 3.64	0.08
Etiology			0.01^*^
Cardiac	10	9	
Non cardiac	31	2	
Initial rhythm			
Asystole	31 (75.6)	7 (63.6)	0.12
VF	7 (17)	3 (27.2)	0.1
PEA	2 (4.8)	1 (9)	0.08
VT	0 (0)	1 (9)	0.22
DC shock	12	4	0.15
Vasopressor infusion	24 (58.5)	4 (36.3)	0.01^*^

Abbreviations: CPR, cardiopulmonary resuscitation; Vf, ventricular fibrillation; PEA, pulseless electrical activity; VT, ventricular tachycardia. Quantitative and qualitative variables were presented as mean ± SD and number (percent), respectively. Significance level was considered as 0.05.

The mean serum cortisol levels of all patients were (61.32 μg/dL; SD ± 6.63) at the beginning of CPR. Baseline and post-ACTH injection mean cortisol levels were significantly higher in females than males (*P* < 0.05).

41 (78.8%), 30 (57.6%) and 6 (11.5%) patients had RAI at 30 and 60 minutes and 24 hours after ACTH stimulation test, respectively.

Patients who died, had higher serum cortisol levels at baseline, 30 and 60 minutes, and 24 hours after cosyntropin test, compared to the survivors (Table 2), but the difference was not statistically significant except to that of minute 60.

**Table 2:** Comparison of serum cortisol level according to the outcome

**Serum cortisol, µg/dL**	**Non-survivors**	**Survivors**	***P***
Baseline	84.25 ± 45.41	49.36 ± 36.46	0.09
30 min post-cosyntropin test	94.04 ± 50.95	57.72 ± 38.90	0.085
60 min post-cosyntropin test	103.23 ± 54.99	62.64 ± 42.36	0.049
24 h post-cosyntropin test	100.05 ± 62.48	97.48 ± 75.90	0.6

Variables were presented as mean ± SD. Significance level was considered as 0.05.

Patients who died, showed more increase in serum cortisol levels at 30 and 60 minutes and 24 hours after cosyntropin test, compared to the survivors (Table 3), but the differences were not statistically significant.

**Table 3:** Comparison of increase in serum cortisol levels according to the outcome

**Increase in serum cortisol levels (µg/dL)**	**Non-survivors**	**Survivors**	***P***
30 min post-cosyntropin test	8.81 ± 22.79	1.00 ± 6.85	0.086
60 min post-cosyntropin test	15.08 ± 24.51	2.17 ± 14.20	0.246
24 h post-cosyntropin test	50.69 ± 52.13	32.81 ± 19.82	0.054

Variables were presented as mean ± SD. Significance level was considered as 0.05.

A model of multivariate logistic regression analysis showed that age and need for vasopressor infusion correlated with mortality (Table 4).

**Table 4:** Results of multivariable regression analysis of factors associated with death

**Characteristics**	**OR**	**CI 95%**	***P***
Sex	0.95	0.77-1.08	0.499
Age	1.1	1.06-1.12	0.03*
Necessity Of vasopressore	2.33	1.40-3.65	0.01*
Primary cardiac rhythm	1.71	0.87-2.44	0.33
CPR duration	1.42	0.88-2.11	0.073
AI according to 30 min post-cosyntropin test	0.99	0.94-1.11	0.27
AI according to 60 min post-cosyntropin test	1.39	0.89-2.08	0.079
AI according to 24 h post-cosyntropin test	1.30	0.90-1.86	0.173

Abbreviations: AI, adrenal insufficiency; OR, Odds ratio; CPR, cardiopulmonary arrest. Significance level was considered as 0.05.

## Discussion

This research showed that patients with higher initial serum cortisol levels or those who had more increase in serum cortisol levels after ACTH stimulation test experienced worse outcome after ROSC, but this is statistically significant only at 60 minutes after ACTH injection.

Previous studies on post CPA patients have shown that vasopressin-dependent shock and impaired adrenal function are common after CPA.^12-14^

Prior studies proved that corticosteroids reverse shock state and improve outcome in patients with septic shock^15,16^; two studies achieved higher ROSC rates with administration of hydrocortisone during CPR.^7,17^

Multiple studies contrary to our study showed that serum cortisol levels are higher in survivors of CPA and impaired adrenal function leads to a dismal outcome.^12^

Some researchers achieved the same results like ours. They found that baseline and post ACTH levels of cortisol were higher in non-survivors; so, they concluded that higher cortisol levels were associated with poorer outcome.^8,9,18^

Conflicting results of these studies may be attributable to some reasons.

First, the level of cortisol is closely related to the severity of underlying disease; so, higher level of cortisol reflects more severity of stress, and it leads to poorer outcome. In CPA as the most critical condition, the higher level of cortisol is expected, particularly in non survivors who are probably exposed to higher level of stress.

Second, although the patients with chronic end-stage disease excluded from the study, there is no information on the cause, severity or duration of the illnesses precipitating the out-of-hospital CPA. Patient’s characteristics and other factors including time since CPA to CPR, duration of CPR and hypotension, time of initial and other sampling for cortisol and time of ACTH test, all may explain the differences among results of similar studies.

The most important point is whether the previous definition of normal basal serum cortisol and RAI (incremental response to ACTH <9 μg/dL) are applicable in the context of ROSC upon CPA or not.

CPA as the most critical illness with sudden multiple organ circulatory collapse and need to special drugs with multiple physiologic effects, is very different from other illnesses.

In this extreme stressful situation, the pituitary adrenal axis is stimulated maximally and may not response further to ACTH stimulation.^19^

On the other hand, in a recent study, Boonen et al^20^ showed ACTH – cortisol dissociation during critical illnesses. They found that cortisol metabolism is decreased in critical illness and results in suppression of cortisol response to ACTH stimulation test; hence, the diagnostic value of adrenal insufficiency is questionable in this situation.

Many researches showed lower serum cortisol levels as a predictor of worse outcome in successfully resuscitated victims of CPA. The new finding of this study is different. So, we need more research for acceptance of this theory to add corticosteroid to the cardiac resuscitation guidelines.

## Limitations

This study had several limitations. Defining adrenal insufficiency as a change in cortisol level of 9 mcg/dL is arbitrary. Although “9” has been defined in some prior studies, the definition of adrenal insufficiency by ACTH stimulation test in sick/critically ill individuals is controversial and not universally applicable. Also, we excluded the patients who did not have successful CPR. They could affect the results of study. Also we do not have any information about acute or chronic clinical status of the patients. More chronic and prolonged critical illness has profound effect on serum cortisol levels.

## Conclusion

Current study couldn’t show the statistically significant difference in initial and post ACTH serum cortisol levels between survivor and non-survivor patients with cardiac arrest who had initial successful CPR, except to that of minute 60. We need more research to find importance of corticosteroid during and after cardiac arrest.

## Acknowledgements

This study was the residency thesis of Dr. Mosaddegh with supervision of Dr. Mofidi by grant of Tehran University of Medical Sciences (TUMS).

## Ethical approval

This study was approved by medical ethics committee of Tehran University of Medical Sciences (TUMS).

## Competing interests

Authors declare no conflict of interests in this study.

## References

[1] Meaney PA, Nadkarni VM, Kern KB, Indik JH, Halperin HR, Berg RA (2010). Rhythms and outcomes of adult in-hospital cardiac arrest. Crit Care Med.

[2] Cooper MS, Stewart PM (2003). Corticosteroid insufficiency in acutely ill patients. N Engl J Med.

[3] Annane D, Cavaillon JM (2003). Corticosteroids in sepsis: from bench to bedside?. Shock.

[4] Bollaert PE, Fieux F, Charpentier C, Lévy B (2003). Baseline cortisol levels, cortisol response to corticotropin, and prognosis in late septic shock. Shock.

[5] Gonzalez H, Nardi O, Annane D (2006). Relative adrenal failure in the ICU: an identifiable problem requiring treatment. Crit Care Clin.

[6] Tavakoli N, Bidari A, Shams Vahdati S (2012). Serum cortisol levels as a predictor of neurologic survival in successfully resuscitated victims of cardiopulmonary arrest. J Cardiovasc Thorac Res.

[7] Tsai MS, Huang CH, Chang WT, Chen WJ, Hsu CY, Hsieh CC (2007). The effect of hydrocortisone on the outcome of out-of-hospital cardiac arrest patients: a pilot study. Am J Emerg Med.

[8] Hékimian G, Baugnon T, Thuong M, Monchi M, Dabbane H, Jaby D (2004). Cortisol levels and adrenal reserve after successful cardiac arrest resuscitation. Shock.

[9] de Jong MF, Beishuizen A, de Jong MJ, Girbes AR, Groeneveld AB (2008). The pituitary–adrenal axis is activated more in non-survivors than in survivors of cardiac arrest, irrespective of therapeutic hypothermia. Resuscitation.

[10] Tordjman K1, Jaffe A, Grazas N, Apter C, Stern N (1995). The role of the low dose (1 microgram) adrenocorticotropin test in the evaluation of patients with pituitary diseases. J Ciin Endocrinol Metab.

[11] Annane D, Sébille V, Charpentier C, Bollaert PE, François B, Korach JM (2002). Effect of treatment with low doses of hydrocortisone and fludrocortisone on mortality in patients with septic shock. JAMA.

[12] Miller JB, Donnino MW, Rogan M, Goyal N (2008). Relative adrenal insufficiency in post-cardiac arrest shock is under-recognized. Resuscitation.

[13] Pene F, Hyvernat H, Mallet V, Cariou A, Carli P, Spaulding C (2005). Prognostic value of relative adrenal insufficiency after out-of-hospital cardiac arrest. Intensive Care Med.

[14] Kim JJ, Lim YS, Shin JH, Yang HJ, Kim JK, Hyun SY (2006). Relative adrenal insufficiency after cardiac arrest: Impact on postresuscitation disease outcome. Am J Emerg Med.

[15] Fernández J, Escorsell A, Zabalza M, Felipe V, Navasa M, Mas A (2006). Adrenal insufficiency in patients with cirrhosis and septic shock: Effect of treatment with hydrocortisone on survival. Hepatology.

[16] Jacobi J (2006). Corticosteroid replacement in critically ill patients. Crit Care Clin.

[17] Mentzelopoulos SD, Zakynthinos SG, Tzoufi M, Katsios N, Papastylianou A, Gkisioti S (2009). Vasopressin, epinephrine, and corticosteroids for in-hospital cardiac arrest. Arch Intern Med.

[18] Annane D, Sébille V, Troché G, Raphaël JC, Gajdos P, Bellissant E (2000). A 3-level prognostic classification in septic shock based on cortisol levels and cortisol response to corticotropin. JAMA.

[19] Kim JJ, Hyun SY, Hwang SY, Jung YB, Shin JH, Lim YS (2011). Hormonal responses upon return of spontaneous circulation after cardiac arrest: a retrospective cohort study. Critical Care.

[20] Boonen E, Vervenne H, Meersseman P, Andrew R, Mortier L, Declercq PE (2013). Reduced cortisol metabolism during critical illness. N Engl J Med.

